# Formation of a Rectus Sheath Hematoma Secondary to COPD Exacerbation While Taking Dual Antiplatelet Therapy

**DOI:** 10.7759/cureus.18821

**Published:** 2021-10-16

**Authors:** Nicholas Salem, Taylor Sharpe, Arminder Singh, Manoj Bhandari

**Affiliations:** 1 Cardiology, Campbell University School of Osteopathic Medicine, Fayetteville, USA; 2 Internal Medicine, Cape Fear Valley Medical Center, Fayetteville, USA; 3 Cardiolgy, Cape Fear Valley Medical Center, Fayetteville, USA

**Keywords:** chronic obstructive pulmonary disease (copd), dual antiplatelet therapy (dapt), ultrasound (us), chronic cough, rectus sheath hematoma

## Abstract

Rectus sheath hematomas can occur due to nontraumatic increases in abdominal pressure from respiratory disease such as chronic obstructive pulmonary disease (COPD). This case study describes a 59-year-old male who was on dual antiplatelet therapy after a right coronary percutaneous intervention for acute coronary syndrome. He developed abdominal pain and ecchymosis on dual antiplatelets and was found to have a rectus sheath hematoma. The hematoma resolved with conservative care and did not require surgical intervention. The etiology of rectus sheath hematoma is thought to be due to coughing spells from chronic obstructive pulmonary disease exacerbation while taking dual antiplatelet therapy. Cases of rectus sheath hematomas continue to emerge in the literature with similar patient histories, and we should be cognizant of this possible complication in patients with chronic coughing.

## Introduction

Various layers of abdominal fascial tissue form the rectus sheath, which surrounds the rectus abdominis muscles and contains the inferior epigastric arteries. When excess intra-abdominal pressure is present, commonly occurring from pathologies of chronic coughing such as chronic obstructive pulmonary disease (COPD), and the patient is in a hypocoagulable state, there is an increased risk that these arteries may bleed. Rectus sheath hematomas form in this scenario, and the below case presentation is a detailed description of this pathology.

## Case presentation

**Figure 1:**
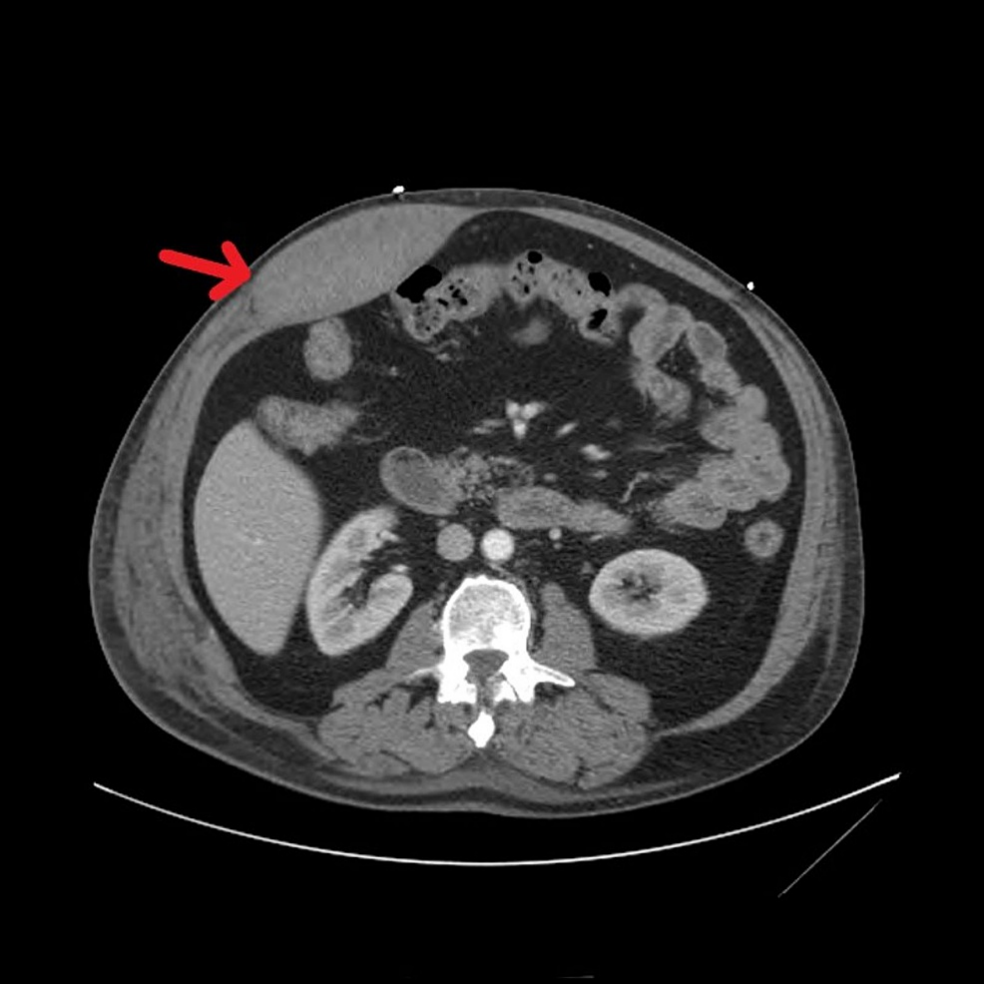
Computerized tomography findings demonstrating a rectus sheath hematoma between the anterior and posterior aspects of the rectus sheath fascia (red arrow).

A 59-year-old male with a past medical history of hypertension, hyperlipidemia, chronic pain taking opioids, chronic obstructive pulmonary disease, gastroesophageal disease, and recently diagnosed coronary artery disease status post stent placement in the setting of acute coronary syndrome, presented for a follow-up cardiology appointment after a recent visit to the emergency department for coughing spells and right-sided flank pain. Of note, eight months prior, the patient was found to have critical stenosis of the distal right coronary artery when he was admitted to the hospital with acute coronary syndrome and was treated with percutaneous coronary intervention and medical management. His medical therapy included guideline therapy of atorvastatin, lisinopril, metoprolol succinate, and dual antiplatelet therapy consisting of aspirin and ticagrelor initially and changed to clopidogrel during outpatient follow-up due to cost. The patient had multiple coughing spells secondary to chronic obstructive pulmonary disease exacerbations before his presentation of flank pain. He had computed tomography (CT) imaging of the abdomen that revealed a 12 × 4.5 × 12 cm hematoma within the rectus sheath musculature (Figure 1) and was discharged with advice to follow up on conservative management.

**Figure 2:**
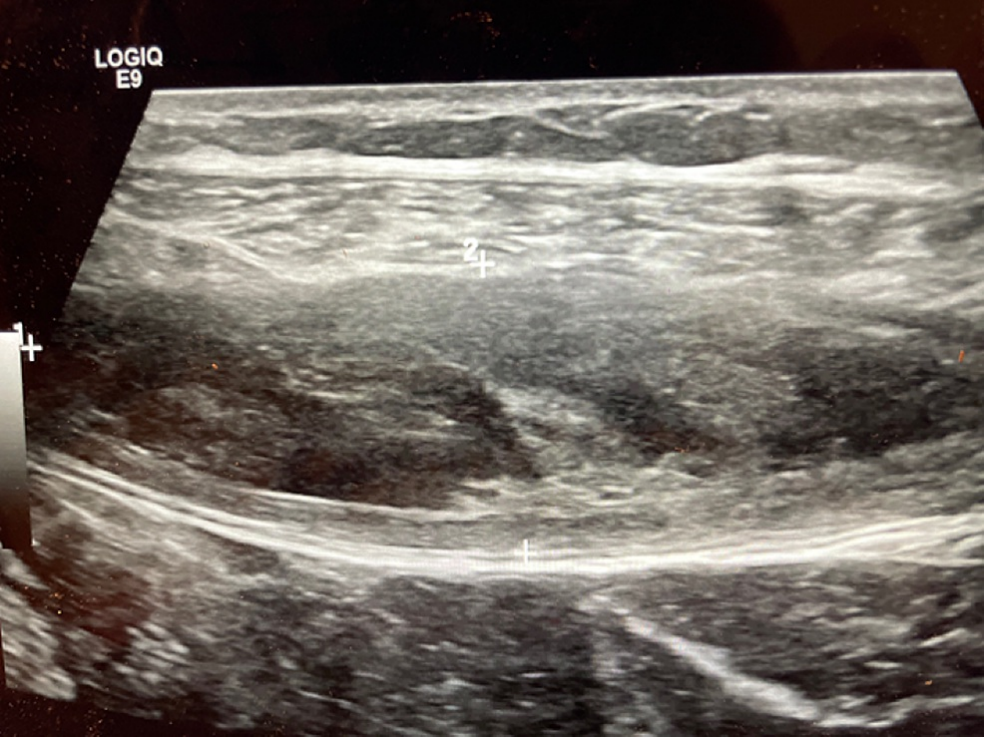
Ultrasonography findings demonstrating a rectus sheath hematoma between the anterior and posterior aspects of the rectus sheath fascia.

His aspirin was stopped, and he continued clopidogrel only on his medication review after the ED visit. He also met with his primary care physician during which ultrasound imaging revealed a decreasing hematoma of 7 × 2 × 5 cm (Figure 2).

During his primary care visit, his COPD treatment was optimized, and he was continued on conservative management for the resolving hematoma. During follow-up with his cardiologist, there was minimal ecchymosis, as compared with the ecchymosis that prompted his initial presentation to the emergency department. His lisinopril was also changed to losartan, and he was advised on the benefits of vaping cessation. The hematoma completely resolved, and he is solely treated with aspirin therapy. He is closely followed by his primary care physician and cardiologist and has not had any recurrence or bleeding for almost 18 months since the development of a rectus sheath hematoma.

## Discussion

Dual antiplatelet therapy consisting of aspirin and clopidogrel has been studied through multiple large clinical trials that have shown in NSTEMI cohorts to reduce future cardiovascular events, nonfatal myocardial infarction, stroke, severe ischemia, heart failure, and repeat revascularization procedures [1,2]. However, there were significant increases in both major and minor bleeding episodes seen during these studies. These findings were further studied in a subgroup of the CURE trial, which demonstrated dual antiplatelet therapy prior to percutaneous intervention and continued for 3-12 months improved outcomes of myocardial infarction and urgent revascularization events with only increases in minor bleeding [3]. However, in this trial, there was no standardized duration of clopidogrel dual therapy, which raised the question answered by the CREDO trial. This trial found that, when a loading dose was administered six hours prior to percutaneous intervention and dual antiplatelet therapy is continued for one year, there was a reduction in myocardial infarction, death, and revascularization events [4]. This was further evaluated by retrospective studies analyzing the use of dual antiplatelets for one year in bare metal and drug-eluting stents, showing a decrease in death and myocardial infarction, more so in bare metal stents compared with drug-eluting stents [5]. For these reasons, dual antiplatelet therapy has become a class 1B recommendation according to AHA guidelines following NSTEMI [6].

Dual antiplatelet therapy has shown to reduce vascular complications in a patient population post acute coronary syndrome; however, this therapy does increase bleeding risks. There are numerous cases of rectus sheath hematomas developing in patients on dual antiplatelet therapy treatment and on new oral anticoagulation alone [7-9]. In a review of rectus sheath hematoma, half were found to be due to nonsurgical abdominal wall trauma, of which the most common cause was coughing [10]. This is directly relevant to our case presentation, as our patient presented to outpatient follow-up coughing and with a past medical history of chronic obstructive pulmonary disease with ongoing vaping use. We should also be aware of this complication when the patient reports abdominal pain without change in bowel habits, following coughing spells while taking antiplatelets and anticoagulation. This may be more relevant now during the COVID-19 pandemic when patients may not seek immediate care or have immediate access due to the emerging social difficulties that the COVID-19 pandemic has presented on patients and the healthcare system. 

## Conclusions

In conclusion, the authors have described a case of the formation of a rectus sheath hematoma due to increased intra-abdominal pressure from COPD in a patient in a hypocoagulable state from taking dual antiplatelet therapy according to AHA guidelines. The authors seek to add this case to the literature due to the common usage of dual antiplatelet therapy and the common prevalence of COPD so that other healthcare providers become familiar with an uncommon pathology. This is an important pathology to bring to attention as rectus sheath hematomas can become life-threatening depending on the degree of blood loss. Fortunately, in this case, the patient’s pathology was rapidly identified and eventually self-resolved with appropriate conservative management.
